# Socioeconomic and Ecological Factors Influencing *Aedes aegypti* Prevalence, Abundance, and Distribution in Dhaka, Bangladesh

**DOI:** 10.4269/ajtmh.15-0639

**Published:** 2016-06-01

**Authors:** Parnali Dhar-Chowdhury, C. Emdad Haque, Robbin Lindsay, Shakhawat Hossain

**Affiliations:** Natural Resources Institute, University of Manitoba, Winnipeg, Canada; National Microbiology Laboratory, Public Health Agency of Canada, Winnipeg, Canada; Department of Mathematics and Statistics, University of Winnipeg, Winnipeg, Canada

## Abstract

This study examined household risk factors and prevalence, abundance, and distribution of immature *Aedes aegypti* and *Aedes albopictus*, and their association with socioeconomic and ecological factors at urban zonal and household levels in the city of Dhaka, Bangladesh. During the 2011 monsoon, 826 households in 12 randomly selected administrative wards were surveyed for vector mosquitoes. Results revealed that the abundance and distribution of immature *Ae*. *aegypti* and *Ae*. *albopictus*, and pupae-per-person indices did not vary significantly among the zones with varied socioeconomic status. Of 35 different types of identified wet containers, 30 were infested, and among the 23 pupae-positive container types, nine were defined as the “most productive” for pupae including: disposable plastic containers (12.2% of 550), sealable plastic barrels (12.0%), tires (10.4%), abandoned plastic buckets (9.6%), flower tub and trays (8.5%), refrigerator trays (6.5%), plastic bottles (6.4%), clay pots (4.9%), and water tanks (1.6%). When the function of the containers was assessed, ornamental, discarded, and household repairing and reconstruction-related container categories were found significantly associated with the number of pupae in the households. The purpose of storing water and income variables were significant predictors of possession of containers that were infested by vector mosquitoes.

## Introduction

Worldwide, more than 50 million dengue virus (DENV) infections occur each year, including 500,000 hospitalizations for dengue hemorrhagic fever (DHF), with the case fatality rate exceeding 5% in some areas.^1^,^2^ However, others estimate the size of annual dengue infection globally to be much larger—390 million, of which 90 million manifest symptoms of varying levels of clinical or subclinical severity.^3^ DENVs are transmitted to people through the bite of container-inhabiting mosquitoes *Aedes aegypti* and *Aedes albopictus*.^2^ Because no antiviral drugs and vaccines are commercially available for DENV, vector control is the primary means to reduce dengue transmission and there are relatively few examples in the literature where dengue outbreaks have been successfully controlled through implementation of vector control measures. In a pilot project in Singapore in the late 1960s, the development of a vector control system, based on entomological surveillance and larval source reduction, resulted in the reduction of the *Ae*. *aegypti* population from 16 to 2% in a 3-month period, as measured by the premises index.^4^,^5^ Guzman and Kouri^6^ observed successful vector eradication campaigns starting in the mid-1940s through larval control in domestic water storage containers and use of insecticides,^7^ and most countries in the Central and South American regions were free of the vector. However, these regions were reinfested by *Ae*. *aegypti* during the 1960s and 1970s.^6^

Often others have observed that the wholesale use of insecticides has nominal impact on the overall vector population and dengue incidence rates.^7^,^8^ Elimination of larval habitats (i.e., source reduction) from the domestic and peridomestic environments is often recommended as a simple and effective alternative method to manage dengue vector populations.^7^,^9^–^11^ Transmission rates can also be dramatically reduced on a theoretical basis, when a reasonable proportion of productive larval development sites are repeatedly treated (rather than permanently eliminated) with nonrepellent larvicides.^12^ Quantifying the variety of larval development sites (i.e., containers) used by vector mosquitoes and how their use changes over time, in a setting of unprecedented pace of change in the cities of the developing world, are therefore essential components required to formulate effective vector control programs. This is quite applicable to Dhaka, Bangladesh—one of the fastest growing cities in the world,^13^,^14^ where a major dengue outbreak occurred in 2000 and dengue cases have been reported annually since, and dengue fever and DHF continued to be a major public health threat.^2^,^15^

In this research, we applied the term mosquito “abundance” to refer to a certain calculated number of mosquitoes per unit of measurement such as person, or area (e.g., pupae-per-person at the household premises); “distribution” to denote proportion of households infested, reflecting how they are located in various sites; and “prevalence” to refer to the proportion of households or containers infested with immature mosquitoes. Containers were considered “productive” in terms of their ability to support immature stages and ultimately produce adult female vectors. Several indicators have been used to quantify the prevalence of dengue vectors (e.g., *Stegomyia* indices, estimates of adult vector density, and use of oviposition indices); however, establishing robust indicators for delineating outbreak risk and the threshold level for outbreak prevention have not yet been very successful.^16^ The *Stegomyia* indices considered in this research included the house index (HI) defined as the percentage of houses infested with larvae and/or pupae of vector mosquitoes, container index (CI) defined as the percentage of water-holding containers infested with larvae and/or pupae, and Breteau index (BI) defined as the number of positive (infested) containers per 100 houses inspected.^1^

More recently, surveillance for pupae of dengue vectors has been recognized as a useful tool for measuring dengue outbreak risk, mainly because the abundance of *Ae*. *aegypti* pupae serve as an effective proxy for the number of female mosquitoes.^17^–^19^ In addition, several urban studies have demonstrated that most *Ae*. *aegypti* pupae were produced in only a few types of containers,^19^–^21^ and as a result, eliminating or treating the “most productive containers” could dramatically improve the efficacy of vector control programs. The concept of most productive containers is used widely,^19^,^21^,^22^ and these “key” containers are identified by determining the relative contribution that a particular container type makes to the overall production of *Ae*. *aegypti* and *Ae*. *albopictus* pupae. Worldwide, very few studies have examined how the dynamics of dengue vector populations are influenced by social and local ecological factors in cities of the tropical regions.^11^,^23^,^24^ In Bangladesh, thus far only two studies^15^,^25^ examined the spatial distribution and prevalence of *Aedes* vectors by households in the city of Dhaka; however, these studies did not consider pupal productivity (PP).

The socioeconomic status (SES) of both urban zones (areal and landscape characteristics emphasis) and household residents (homestead premise emphasis) has been found to be significant factors in influencing the number and types of mosquito-infested containers in and around households.^26^–^30^ However, some studies, especially in Latin America,^29^,^31^–^33^ questioned the hypothesis concerning the role of SES in the abundance of *Ae*. *aegypti* as they found no significant variation among SES zones in the cities studied. These contrary findings on the role of SES underscore the need for further research on the role it may play on the abundance and distribution of dengue vectors.

To address these gaps, the specific objectives of this research were to examine a number of questions regarding the patterns in *Ae*. *aegypti* and *Ae. albopictus* prevalence, abundance, and distribution within containers from households located in three distinct urban zones of Dhaka with different SES. These included 1) whether the distribution and prevalence of *Ae. aegypti* and *Ae. albopictus*, represented by standard *Stegomyia* indices, differ significantly among the various urban zones with “low,” “medium,” and “high” SES; 2) to identify “positive” (ones infested with vector mosquitoes) containers to gain a better understanding of the utilization of different “types” of containers by *Ae. aegypti* and *Ae. albopictus*; and 3) to determine whether *Ae. aegypti* PP was significantly associated with various container types within households.

The second set of questions inquired specifically about, 4) the association of *Ae. aegypti* PP with the “functional categories” (FCs) of positive containers; 5) the patterns in the prevalence of *Ae. aegypti* pupae in various container types and to investigate whether containers housing pupae were clustered in certain locations within the household premises (i.e., indoor and outdoor ecological settings); and 6) whether the household socioeconomic, infrastructural, and human behavioral factors are significantly associated with the possession of positive containers.

## Materials and Methods

### Study area, design, and population.

**Figure 1.**
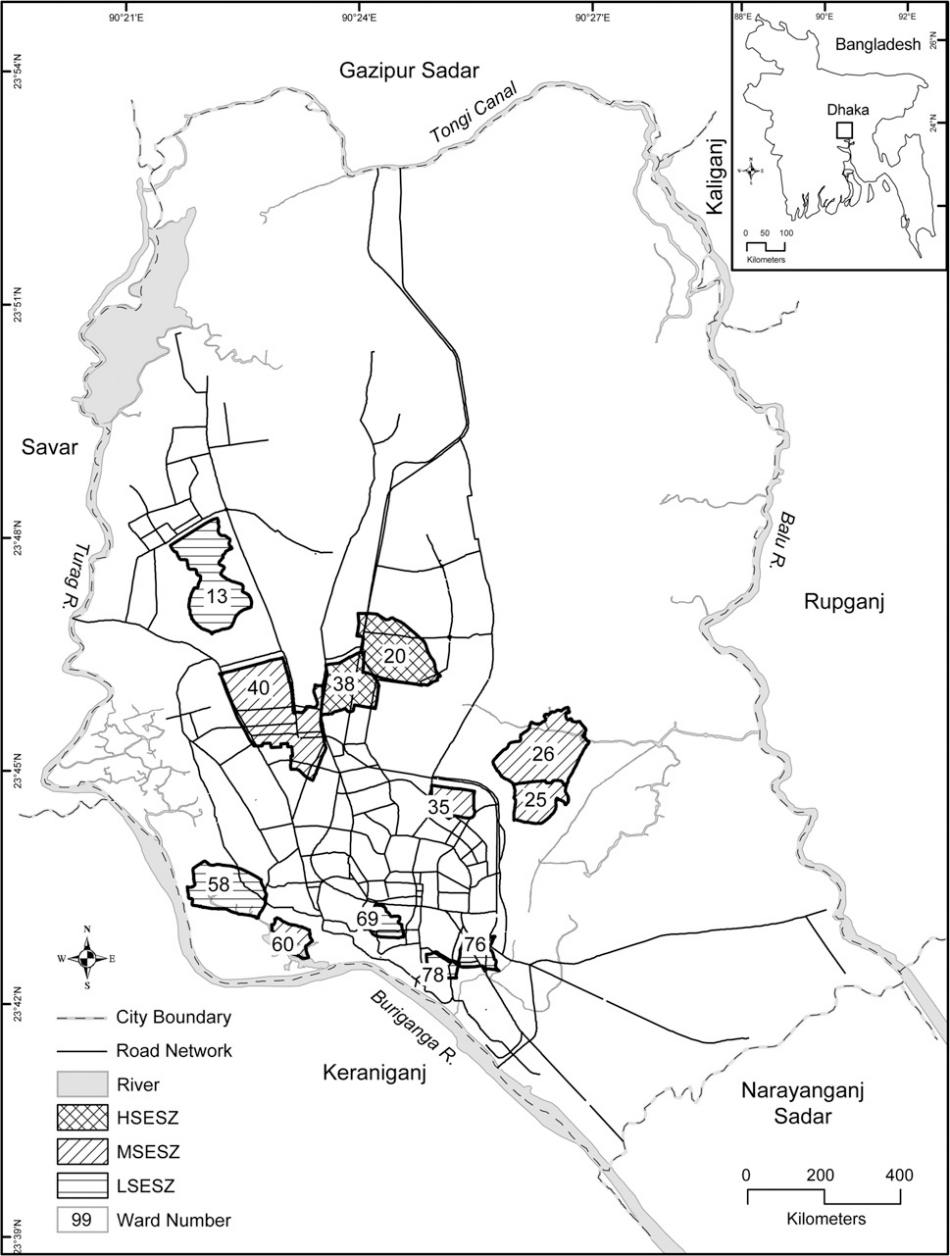
Location of study area and distribution of the 12 selected wards in the city of Dhaka, Bangladesh, by socioeconomic status (SES) zone. HSESZ = high SES zone; LSESZ = low SES zone; MSESZ = medium SES zone.

Dhaka—the capital of Bangladesh—is the ninth largest city in the world,^34^ and with more than 12 million (2011 census) people, it is one of the most densely populated cities in the world. The city experiences a hot, wet, and humid tropical climate, with monthly mean temperature varying between 20°C (68°F) in January and 32°C (90°F) in May.^35^ It is spread over an area of 360 km^2^, which is administratively divided into 90 wards (a “ward” is the local administrative unit of the city corporation). Using the Delphi method,^36^ considering the socioeconomic differentials among these wards, and eight specific criteria (i.e., municipal property tax, property market value, rate of property rent, proximity of types of markets and shopping areas, types of building structure, proximity to public services, state of infrastructure, and state of transport in each ward), we categorized 36 wards as low (LSES), 40 wards as medium (MSES), and 14 wards as high (HSES) zones. Probability proportional sampling method was applied to 12 randomly selected wards (12/90; 13%) for the entomological survey of household premises, resulting in five LSES wards, five MSES wards, and two HSES wards (Figure 1

). A random sample of 100 households from each selected ward was targeted, to attain an overall sample size of 1,200 households. Within each ward, a spatial randomization procedure was followed by drawing grid cells on a map of the wards and selecting a total of 100 households using a random number table.

Considering that the incidence of dengue is usually highest in August in Dhaka,^37^ entomological surveys to detect larvae and pupae of *Ae. aegypti* and *Ae. albopictus* were carried out during the monsoon season (last week of July to the first week of August) in 2011. The mean precipitation and temperature for the months of July and August of 2011 with corresponding long-term parameters for the 1985–2014 period were compared with by testing the meteorological “anomaly (Supplemental Figures 1 and 2).” We inferred that although July of 2011 was nominally warmer (by 0.4°C) and drier (by 0.75 mm of rainfall) and August was nominally cooler (by 0.5°C) and wetter (by 3.45 mm) relative to long-term means, these 2011 summer months followed typical monsoon mean patterns (i.e., these parameters were within ± 1σ). Multiple teams of field entomologists inspected households in different wards of the three SES zones simultaneously to ensure aggregation and comparability.^18^ Informed consent was requested from householders before conducting each household survey.

### Data collection techniques.

All containers located inside as well as within a 50-m radius of the household were inspected for immature mosquitoes. Any water-filled container where water was stored or accumulated for more than 3 days, as applied by a number of previous entomological surveys in the region,^22^,^38^ was considered a “wet container” and all wet containers within a household were counted. The “type” (determined by the common use of water-holding vessels by the locales) of all wet containers was recorded for each individual item. “Positive containers” were those found in the household, which were infested with *Ae. aegypti* and/or *Ae. albopictus* larvae and/or pupae. Notably, due to lack of access and ownership of multistoried properties, known “cryptic” mosquito development sites such as, rooftop water tanks, sump pumps, and underground cisterns could not be sampled and inspected during this survey.

The *Stegomyia* indices (HI, CI, and BI) were estimated to quantify the difference in vector mosquito prevalence and abundance among SES zones. In this research, indices regarding PP^39^,^40^ were measured in terms of total pupae count per container or per house or per person. Appropriate confidence intervals were calculated using the proportion of HI, CI, and BI:

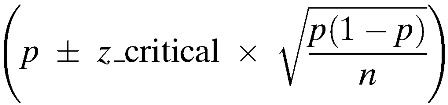
where *p* is the proportion. The contents of most wet containers were carefully inspected by emptying them onto white enamel pans. For larger containers, larvae or pupae were collected by dipping sampling and the total number of each instar per container was estimated. Field entomologists visually inspected large containers and then performed five sequential dips to determine the presence or abundance of immature mosquitoes. When containers were infested with fewer than 10 pupae or larvae, all were transferred to a 10-mL Falcon tube; however, when larger numbers were present, only a subset of larvae and pupae were collected. Estimating the number of pupae based on a subset generally would have underestimated the number of pupae per person; however, as relatively few containers in the study area had more than 10 pupae, the likelihood of such underestimation was minimal. Field entomologists estimated the total number of larvae and pupae present in large containers by multiplying the number of immatures found by sequential dipping with the estimated volume of water in the infested containers. Tubes containing larvae or pupae were transported to the laboratory at North South University (Dhaka, Bangladesh). Once in the laboratory, larvae were killed by submerging them in formaldehyde and species determination for larvae was performed using dissecting microscopes and the taxonomic keys of Consoli and de-Oliveira.^41^ All collected pupae were reared to the adult stage in the laboratory, before species determination. The pupae per person index (PPI) was calculated by dividing the total number of *Ae. aegypti* pupae collected in a household premise by the total number of persons who usually “reside and sleep” in the same household.^42^

The information regarding household population was collected during the socioeconomic and demographic survey, which was carried out by interviewing the household heads, simultaneously with the entomological surveys. Using a structured questionnaire, primary household-level data on socioeconomic (e.g., income, assets) and demographic characteristics of the members living in each household, water supply system to the household, water use, and waste disposal and management were collected. The questions were asked in the local language (i.e., Bangla) and the responses were translated to English by experienced research fellows.

### Statistical analyses.

All data were compiled and analyzed using SAS (SAS Institute Inc., Cary, NC) and IBM SPSS version 21 (IBM Corp., Armonk, NY). Our estimates are robust as the sample size was relatively large and there was no misclassification in the response. We used sandwich variance estimates to calculate the confidence intervals of the estimates. Descriptive statistics were used to summarize the data with confidence limit mean ± standard error. Corresponding to the first set of specific objectives, a hypothesis whether the prevalence and distribution of *Ae. aegypti* and *Ae. albopictus*, represented through *Stegomyia* indices, differ significantly among the various urban zones with “low,” “medium,” and “high” SES was tested. We used the χ^2^ test of homogeneity to make inference about *Stegomyia* indices among urban zones with varying SES. Spearman's correlation was used to evaluate the strength of the relationship between container abundance and PP of each type of container. The χ^2^ test was used to evaluate whether there was a significant difference in the distribution of positive containers within households among the SES zones, and the Kruskal–Wallis test was used to determine whether there was a significant difference in the distribution of most productive containers within households among the SES zones. The χ^2^ test was used to determine whether the distribution of *Ae. aegypti* pupae in the sampled households varied significantly in accordance with container types.

Regarding the second set of questions, Poisson regression analysis, cluster analysis, and logistic regression analysis were applied. Considering the diversity of types of containers and to minimize the variability among the containers, we categorized them based on five household FCs. As a result, containers were functionally classified as those used for 1) household chores (A), 2) ornamental purposes (B), 3) amenities (C), 4) discarded containers (D), and 5) repairing and reconstruction purposes (E). The association between the FCs of positive containers and *Ae. aegypti* PP was determined. A bivariate regression analysis between PP and each individual explanatory variable was carried out to examine further using the cutoff *P* value < 0.25. On the basis of the results, a multivariable Poisson regression model, with stepwise method, was formulated to assess the relationship between the FCs of containers and the PP. The GENMOD SAS procedure (SAS Institute Inc., Cary, NC) was used to fit this model. The risk ratios (RRs) with 95% confidence intervals were calculated to measure the effects. The above methodologies were used to find the relationship between containers (positive and negative) and PP.

To delineate patterns in the prevalence of *Ae. aegypti* pupae in various container types and to investigate whether pupae-positive containers were clustered in locations within the household premises (i.e., indoor and outdoor ecological settings), we applied a two-step cluster analysis.^19^,^43^,^44^ Following the formulation of “natural groupings” or “clusters” of the pupae-positive containers in terms of the sources of water (i.e., rain water, tap water, or both), location under sunshade, proximity to vegetation, statistics regarding *Ae. aegypti* pupae per container for each cluster were calculated. The variable of sunshade was quantified in terms of the proportion of the containers shaded by visual inspection (i.e., full, half, quarter). A two-tailed *t* test was used to compare mean pupae per container across the clusters. Finally, a logistic regression model was used to determine whether household SES, water supply, waste disposal, and water storage were associated with the presence of larvae, pupae, or both in water containers in households.

### Ethics statement.

The study was approved by the Bangladesh Medical Research Council and the Joint Faculty Research Ethics Board at the University of Manitoba. The purpose and objectives were explained to the head of each household and his/her informed consent was obtained orally to inspect the household premises for the presence of dengue vectors and to obtain responses to a socioeconomic and demographic questionnaire.

## Results

With an overall survey response rate of 69%, a total of 826 households were inspected (Table 1). When the residents of the randomly selected household refused to participate or were not home, alternative households were selected when time permitted. Because the survey response rate met the minimum required response rate (more than 50%)^45^,^46^ and achieved 69% overall, the likelihood of “nonresponse bias” due to variation in response rate by SES zone is minimum.^47^

### Patterns in *Ae. aegypti* and *Ae. albopictus* abundance and distribution, types of positive containers, and the role of SES of urban zones.

#### Abundance of immature Ae. aegypti and Ae. albopictus by SES zone.

A total of 4,217 immature *Ae. aegypti* and *Ae. albopictus* (3,667 larvae of which all were III or IV instar, and 550 pupae) were collected from 1,278 wet containers found within 221 household premises located within the three SES zones. In the larval counts, 86% were *Ae. aegypti* and 14% were *Ae. albopictus*. All pupae (*N* = 550; 205 male and 345 female) found during the inspection were *Ae. aegypti* and these pupae were collected from 97 containers (7%). Interestingly, although a small number of *Ae. albopictus* larvae were collected during the surveys, none of the collected pupa that were reared to the adult stage in the laboratory were *Ae. albopictus.* This finding led the analysis to focus on *Ae. aegypti* in the remainder sections of this paper. Distribution and prevalence of vector mosquitoes were described by the traditional *Stegomyia* indices and overall 59.6 positive containers were observed per 100 households inspected (Table 2). Overall, the mean PPI was 0.58; in LSES zone it was 0.53, in MSES zone it was 0.55, and in HSES zone it was 0.62 (Table 2). The *Stegomyia* indices did not vary significantly among the SES zones (HI χ^2^ = 3.6, degrees of freedom [df] = 2, *P* = 0.166; CI χ^2^ = 1.3, df = 2, *P* = 0.52). We therefore conclude that the prevalence and distribution of immature *Ae. aegypti* and *Ae. albopictus* do not vary significantly among the SES zones.

#### Main “types” of positive containers, most productive containers, and abundance of positive containers by SES zone.

**Figure 2.**
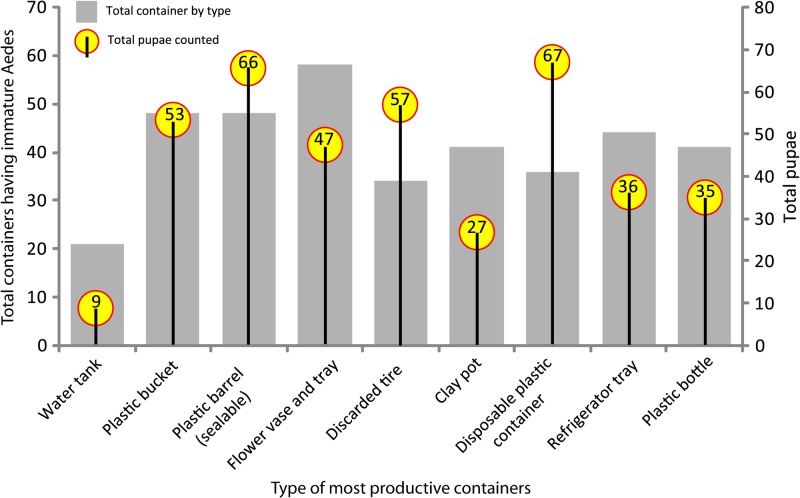
Number of most productive containers (in terms of their relative contribution to total immature *Aedes* mosquitoes) by type of containers and total pupae counted in each type of most productive containers.

In total, of 35 different types of wet containers, 30 types (86% of all wet containers) were identified as positive containers (Supplemental Table 1). Among these positive containers, pupae were found in 23 types of containers (77% of all infested containers). The χ^2^ test results revealed that there was no significant difference in the distribution of positive containers in households among the SES zones (χ^2^ = 0.38, df = 2, *P* = 0.8). Altogether, nine container types of the 371 positive containers having immature *Aedes* mosquitoes, produced 78% of the immature *Ae. aegypti* and *Ae. albopictus* (3,291 of 4,221). In addition, 72% of all *Ae. aegypti* pupae (397 of 550) were classified as the most productive containers (Supplemental Table 2). These containers included: disposable plastic containers (12.2% of 550), sealable plastic barrels (12.0%), tires (10.4%), abandoned plastic buckets (9.6%), flower tub and trays (8.5%), refrigerator trays (6.5%), plastic bottles (6.4%), clay pots (4.9%), and water tanks (1.6%) (Figure 2

). A comparison of the patterns in the distribution of the most productive containers, in terms of relative percentage of total pupae collected, by SES zone reveals some variations. However, based on the Kruskal–Wallis test, we inferred that there was no significant difference in the distribution of most productive containers in household premises among the SES zones (χ^2^ = 3.5, df = 2, *P* = 0.17).

In the study area overall, the most abundant positive containers were also the most productive containers in the inspected households, with the exception of “money plant” tub (*Epipremnum aureum*) (Supplemental Table 2). The most abundant positive containers in the LSES zone were clay pots, plastic bottles, abandoned plastic buckets, sealable plastic barrels, and refrigerator trays (accounting for 53% of all positive containers); whereas in the MSES zone, flower tubs and trays were the most abundant positive containers (22%); and in the HSES zone, discarded tires were most abundant (20%).

#### Container types and Ae. aegypti PP.

Considering that indicators of *Ae. aegypti* PP are better tools for measuring dengue outbreak risk,^18^ we determined that a Spearman correlation between the rank orders of container abundance (in terms of container type) and *Ae. aegypti* PP was positive and significant (*N* = 23; *r*_s_ = 0.817, *P* < 0.001). A total of 411 pupae were found in the nine most abundant containers; among them, disposable plastic containers had the most pupae (16%) followed by plastic barrels (sealable) (16%), tires (14%), abandoned plastic bucket (13%), and flower tub and tray (12%). These positive containers yielded 53% of all *Ae. aegypti* pupae and represented approximately 18% of all wet containers inspected. The χ^2^ goodness-of-fit test revealed that the pupae distribution by container type was not homogeneous (χ^2^ = 72.5, df = 10, *P* < 0.0001).

### Relationships among *Ae. aegypti* PP, FCs of containers, and household ecological and socioeconomic factors.

#### Relationships between FCs of containers and Ae. aegypti PP: a Poisson regression model.

*Aedes aegypti* PP in household premises (i.e., pupal count) related to the FCs of positive containers can be formulated by the following Poisson regression model:

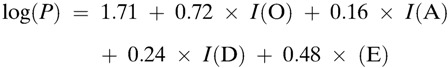
where, I (G) = 1, if G belongs to container category and 0, otherwise. The household chore (A) was assumed as a reference category: P = pupal count; O = ornamental purposes; A = amenities; D = discarded containers; E = repairing and reconstruction purposes.

Stepwise method has revealed that the ornamental, discarded, and household repairing and reconstructionr-related containers were significantly related to the number of *Ae. aegypti* pupae found in household premises (ornamental versus household chore: RR = 2.06 (95% CI: 1.59–2.67), *P* < 0.0001; discarded versus household chore: RR = 1.28 (95% CI: 0.99–1.64), *P* = 0.061, and others versus household chore: RR = 1.62 (95% CI: 1.17–2.24), *P* = 0.0035 (Table 3). More than twice as many *Ae. aegypti* pupae were present on average per household in ornamental container types compared with the household chores containers, after adjusting for all other container types.

#### Patterns in prevalence of Ae. aegypti pupae by container types and their association with household ecological settings.

Two distinctive groups or clusters of positive containers with pupae were produced by the two-step cluster analysis (clusters I and II; Table 4). With 42% of the samples, cluster I was composed of flower tubs and tray, tires, clay pots, and discarded plastic containers. Cluster I had 192 (35%) *Ae. aegypti* pupae (mean pupae per container: 0.94 ± 0.20; Table 4). Indoor containers were predominant in cluster I as 91% of all containers were found under the full shade, indoor or outdoor.

Cluster II included 58% of all containers, consisted mostly of sealable plastic barrels, refrigerator trays, plastic bottles, ceramic pots, water tanks, and houseplant (*E. aureum*) pots. The mean number of pupae per container was 1.25 ± 0.19 (total pupae 358 or 65%; Table 4). Outdoor containers were most abundant in this cluster whereby all were filled with rain water, and 82% had vegetation present nearby. The two-tailed *t* test result revealed that the mean number of pupae per container did not vary significantly between the two clusters (*t* statistic = 0.57, *P* = 0.59).

#### Factors associated with possession of containers by household premises: a logistic regression model.

A multivariable logistical regression model was used to determine whether the household socioeconomic, infrastructural, and behavioral factors are associated with the FC of containers. Results from the stepwise logistic regression method revealed that only the household annual income (χ^2^ = 15.6, df = 8, *P* = 0.0481) and the purpose of water storage (χ^2^ = 11.8, df = 4, *P* = 0.0187) were significantly related to the FC of the containers (Table 5).

It appears that the medium-income group had fewer of the most productive containers than high-income group, provided all combinations of other income groups are constant. This is shown in the estimated odds ratio for B versus A of income variable which was exp(−0.40) = 0.66 (Table 5). The regression model results also revealed that the households that store water for the purpose of cleaning, washing, and showering would have more than three times higher most productive containers than when water was stored for drinking and cooking. This is reflected in the estimated odds ratio for B versus A of purpose of storing water was exp(1.19) = 3.29 (Table 5).

## Discussion

The results of our study provide evidence that *Ae. aegypti* productivity is significantly influenced by household socioeconomic factors, through container ownership and ecological factors in the city of Dhaka, Bangladesh, and they are consistent with the findings of some recent studies.^11^,^23^–^25^ With respect to both indoor and outdoor water containers, as a source of infestation, the findings conform to conclusions of many other investigations in urban areas of the developing world.^11^,^16^,^19^,^21^,^23^–^25^ The underlying reasons for residents to store water and inadvertently create development sites for vector mosquitoes were related to access to piped sources of potable water, as 31% of the city residents do not have access to water mains,^25^ and interruptions in water supply by frequent power failures. We found a statistically significant increase in *Ae. aegypti* in household premises where water was stored for longer periods (i.e., more than 3 days) to cope with the uncertainty in power and water supply services. Locally known as the “load-shedding” of electricity,^48^ the daily intermittent interruptions of power supply has been a common phenomenon for the last few decades.^49^,^50^ As most residents in Dhaka use electrical pumps to draw up water into the roof-top reservoirs, the power supply interruptions, in turn, have resulted in serious disruptions in water supply to households via municipal pipelines. The need to store water (for drinking, taking baths, doing household chores, and other necessary work) in containers of various sizes has thus created a very conducive environment for mosquito development, especially during monsoon seasons.^25^,^50^

In exploring the hypothesis concerning whether most abundant containers were most pupae productive, we registered that it holds true, as disposable plastic containers and sealable plastic barrels had most pupae (32%) among the most abundant nine container types. Because of inaccessibility to water mains or lack of reliability in water supply by the frequent power cuts, an overwhelming majority of the residents tend to store water for household chores and other domestic functions, as well as in temporary water tanks for repairing and reconstructing houses. These findings imply some degree of positive correlation of *Ae. aegypti* PP with dwellers' longer term storing of municipal water due to inadequate urban services as well as with factors related to SES of urban zones (e.g., there were more pupae per person in the high SES zones than others) as well as individual households (e.g., annual income) in Dhaka.^51^ Because most urban residents are unaware that longer term storage of water (in open containers) indoors or accumulation of rainwater in containers outdoors can create development sites for *Aedes* mosquitoes,^51^,^52^ they often keep water for many days and/or overlook water that accumulates in outdoor containers.

Two comparable studies in Thailand and Cuba, which directly examined the density of larval populations in household inspections and socioecological risks, enable us to highlight some insights. The context for these studies, nonetheless, offers different kinds of challenges for controlling dengue, mainly because of variation in abundance of *Ae. aegypti* in the study areas.^11^ It is usually much more difficult to get buy-in for source reduction from household residents when mosquitoes are rarely seen, whereas it might be somewhat easier to convince householders to eliminate standing water when mosquitoes can frequently be observed in and around the home such as in Dhaka. The BI was 154.8 in Thailand; whereas it ranged from 0.11 to 1.32 in central Havana, Cuba; and in Dhaka, Bangladesh, we found it varied between 52.0 and 63.4. The 2000 entomological survey in all of 90 wards by multidonor agencies calculated BI as 24.6.^25^ The observation of moderately high rates of infestation of households throughout Dhaka in the current studies and others^25^ warrant the implementation of community-based mosquito abatement programs, to manage *Ae. aegypti* populations. These programs should focus on source reduction strategies including proper management of water storage containers. In consideration of heterogeneity of household premises within each SES zone, we expected no significant difference among them in terms of *Stegomyia* indices and pupae prevalence. The hypothesis was supported by the results of Kruskal–Wallis tests. Although HSES zone had nominally higher PPI (0.62) relative to MSES zone (0.55) and LSES zone (0.53), these variations were not significant, and likely attributed to variation in human population density in each zone. In Thailand, Barbazan and others^53^ recorded PPI of 0.8 in urban areas, and in Cambodia, Seng and others^16^ observed PPI to range between 1.0 and 4.4. In Dhaka, a relatively lower PPI than urban Thailand and Cambodia is also likely the result of higher population density in Dhaka compared with the other studies.

The classification of the pupae-positive containers, using the two-step cluster analysis, based on ecological variables revealed that most *Ae. aegypti* pupae in the study area were in rain-filled containers outdoors. The most productive containers were those situated in direct exposure to sunlight (i.e., not under shade), which received rainfall directly and with varying (i.e., full or scanty) water volume. The rest of the pupae were in containers used to store water for household chores or houseplant pots. From this classification, we can infer that a significant reduction in the *Ae. aegypti* populations could be achieved through simple changes to the behavior of household residents (e.g., using lids on containers, refraining from putting houseplants indoors, and regular draining of water from refrigerator trays) and management (removing discarded containers and/or placing them so that water does not accumulate) of their household premises in the city of Dhaka.

Most studies on *Ae. aegypti* consider examining various container types in household premises and their effects on pupae productivity but they ignored analysis of the underlying factors that lead to the possession of the different type of containers by householders.^21^,^39^,^54^ Specifically, determination of the degree of association between SES of households and container ownership patterns would enable policy makers to target specific households by SES that possess most productive containers. To the best of our knowledge, our study is the first to assess which explanatory socioeconomic factors affect the possession of different container types by householders; thus this research advanced some new knowledge in this area. A multivariable regression analysis between possession of containers by FC and socioeconomic variables indicated that the amount of household income as well as the purpose of storing water significantly affects the category of containers possessed by householders. Further research should be undertaken to validate the influence of more explanatory socioeconomic variables on the possession of different type of containers by householders.

The scope of this study is limited by several factors. First, given the limited scope of this study, we could collect the entomological data only at a single time point and it was not replicated, so the trends we observed may have been different if we did the surveys over several seasons.^55^,^56^ Surveillance efforts focused primarily on containers within and immediately around households, and the contribution of “other” cryptic mosquito development sites, such as rooftop water tanks, septic tanks, storm sewers or drains, and roof gutters,^57^–^62^ to the production of vector mosquitoes was not accounted for. In some instances, especially during the dry season,^57^ cryptic sites can generate large numbers of *Ae. aegypti* females and these sites should be considered in subsequent studies in Dhaka. As has been reported by others,^63^,^64^ it is difficult to accurately assess the risk of human exposure to DENV infection based on abundance of immature mosquitoes (especially larvae). This is because the thresholds for control are inherently dynamic, often nonlinear, and influenced by a complex suite of other factors such as the level of herd immunity, circulating serotypes, human population density and contact rates between vectors and humans, and ambient temperatures and weather profiles.^65^ Since this study is the first *Ae. aegypti* and *Ae. albopictus* PP study in Dhaka, we expect future research endeavors to attempt to fill in these gaps or address the deficits from an entomological perspective. Second, in consideration of sociodemographic, economic, and infrastructural heterogeneity among the wards, generalization of the estimates and inferences to apply to the context of the city of Dhaka as a whole should be made cautiously. Third, although the representation of the sampled households through random selection ensured robust estimates of the household population of the city, inadequate coverage of cryptic larval development sites has constrained the estimation of *Ae. aegypti* production. Finally, in this study, we focused mainly on household containers and surroundings to assess *Ae. aegypti* and *Ae. albopictus* habitats and excluded other habitats in the urban environment. To capture *Ae. aegypti* and *Ae. albopictus* dynamics fully, future studies will need to examine the phenology and distribution of mosquitoes in non-household environments.

### Policy implications.

Specific policy implications of our study can be summarized under the following topics: we determined that nine types of containers are more significant than others in producing mosquito vectors and targeting these most productive container types is needed to optimize labor efficiency and minimize costs while maximizing vector population reduction.^17^,^20^,^40^ Improving regular electricity and water supply has the potential to reduce dengue risk in Dhaka and in other urban centers of Bangladesh. In addition, urban infrastructure development should be incorporated with social communication campaigns aimed at changing householders' water storage behaviors. Our research has highlighted the ubiquity of *Ae. aegypti* development across the SES zones of the city of Dhaka and impracticality of targeting all potential vector habitats via centralized insecticidal interventions. A two-pronged approach, involving intersectoral (e.g., power and water supply, municipal waste management, infrastructure) authorities from multilevel governments (national, city corporation, local) as well as community-based organizations, would be the best option to control *Aedes* and dengue outbreak in Dhaka. In formulating intervention programs, considerations should be given to the varying effects of disruptions, inefficiencies, and lack of coordination of these services on various socioeconomic classes, as explained elsewhere.^48^–^52^ In addition, the scope of future surveillance efforts should be broadened to account for cryptic mosquito development sites, which can produce a large proportion of the standing crop of vector mosquitoes. Finally, consideration should be given to simultaneously measuring the abundance of the adult mosquitoes as this would serve as a means to account for or identify highly productive cryptic development sites that may have been missed during surveys focused on immature mosquitoes.

## Supplementary Material

Supplemental Datas.

## Figures and Tables

**Table 1:** Distribution of response rate by ward and SES zone, average number of wet containers per household, and average number of immature mosquito positive containers per household by ward and SES zone

Ward no. and name (SES)	No. of households targeted	No. of inspected households	Response rate (%)	No. of wet containers	Average no. of wet container per household	No. of positive containers	Average no. of immature mosquito positive containers per household
LSESZ	500	375	75	560	1.49	235	0.63
Mirpur Pierbag	100	60	60	93	1.55	43	0.72
Hazaribagh	100	69	69	112	1.62	14	0.20
Chankharpul	100	83	83	126	1.52	66	0.80
Sutrapur	100	80	80	137	1.71	80	1.00
Narinda	100	83	83	92	1.11	32	0.39
MSESZ	500	317	63	470	1.48	180	0.57
Goran Khilgaon	100	73	73	93	1.27	22	0.30
Banashree	100	63	63	108	1.71	57	0.90
Malibagh	100	65	65	91	1.40	38	0.58
Monipuripara	100	53	53	93	1.75	42	0.79
Lalbagh	100	63	63	85	1.35	21	0.33
HSESZ	200	134	67	248	1.85	78	0.58
Mahakhali	100	67	67	124	1.85	41	0.61
Nakhalpara	100	67	67	124	1.85	37	0.55
Total (all zones)	1,200	826	69	1,278	1.55	493	0.60

HSESZ = high SES zone; LSESZ = low SES zone; MSESZ = medium SES zone; SES = socioeconomic status.

**Table 2:** *Stegomyia* indices and PPI in three SES zones (ward *N* = 12), Dhaka, 2011

SES zone	HI*	95% Confidence interval	CI*	95% Confidence interval	BI*	95% Confidence interval	Number of collected pupae	PPI
LSESZ (5 wards)	25.3	(16.8–33.8)	37.6	(28.1–47.1)	62.9	(53.4–72.4)	107	0.53
MSESZ (5 wards)	20.6	(12.6–28.5)	31.2	(22.1–40.3)	52.0	(42.2–61.7)	169	0.55
HSESZ (2 wards)	18.6	(11.0–26.2)	28.8	(19.9–37.7)	63.4	(53.9–72.8)	271	0.62
All SES zones	26.7	(23.6–29.7)	32.8	(30.4–35.2)	59.6	(56.3–62.9)	547†	0.58

BI = Breteau index; CI = container index; HI = house index; HSESZ = high SES zone; LSESZ = low SES zone; MSESZ = medium SES zone; PPI = pupae-per-person index; SES = socioeconomic status.

* See Section Materials and Methods for definitions of these indices.

† Three pupae become adults during the transfer from the field to the laboratory.

**Table 3:** Descriptive and bivariate analyses of association between container categories (functional) and socioeconomic/infrastructural variables (*N* = 163), Dhaka, 2011

Socioeconomic/infrastructural variable	Total pupae-positive containers (*N* = 163) *n* (%)	Containers category based on functional types [*n* (%)]					*P* value
		Household chores (A), *N* = 73	Ornamental (B) *N* = 20	Amenities (C) *N* = 22	Discarded (D) *N* = 37	Repairing and reconstruction (E) *N* = 11	
Educational status (household head)							
Primary	73 (44.78)	27 (36.98)	10 (50.00)	10 (45.45)	21 (56.76)	5 (45.45)	0.575
Secondary	57 (34.97)	26 (35.62)	6 (30.00)	9 (40.91)	11 (29.73)	5 (45.45)	
Graduate	33 (20.25)	20 (27.40)	4 (20.00)	3 (13.64)	5 (13.51)	1 (9.10)	
Occupation (household head)							
Housewife/unemployed	54 (33.13)	27 (36.98)	7 (35.00)	3 (13.64)	12 (32.43)	5 (45.46)	0.47
Business	43 (26.38)	18 (24.66)	3 (15.00)	9 (40.91)	10 (27.03)	3 (27.27)	
Service	66 (40.49)	28 (38.36)	10 (50.00)	10 (45.45)	15 (40.54)	3 (27.27)	
Income (annual household) (in Taka)†							
Low (< 30K)	51 (32.08)	34 (46.57)	6 (30.00)	5 (22.73)	5 (15.15)	1 (9.09)	0.0481*
Medium (30–70K)	54 (33.96)	22 (30.14)	6 (30.00)	9 (40.91)	15 (45.45)	2 (18.18)	
High (> 70K)	54 (33.96)	17 (23.29)	8 (40.00)	8 (36.36)	13 (39.40)	8 (72.73)	
Water supply (provisions to household)							
Piped water	123 (75.46)	53 (72.60)	16 (80.00)	16 (72.73)	28 (75.68)	10 (90.91)	0.79
Tube wells and others	40 (24.54)	20 (27.40)	4 (20.00)	6 (27.27)	9 (24.32)	1 (9.09)	
Waste disposal							
Municipal/private pick up	143 (87.73)	62 (84.93)	18 (90.00)	19 (86.36)	34 (91.89)	10 (90.91)	0.899
Open disposal	20 (12.27)	11 (15.07)	2 (10.00)	3 (13.64)	3 (8.11)	1 (9.09)	
Purpose of storing water in household							
Drinking and cooking	70 (42.94)	42 (57.53)	6 (30.00)	7 (31.82)	11 (29.73)	4 (36.36)	0.0187*
Cleaning, washing, showers	93 (57.06)	31 (42.47)	14 (70.00)	15 (68.18)	26 (70.27)	7 (63.4)	

* Significant at *P* < 0.05 level.

† Collapsed six income categories into three categories for valid statistical analysis. Gross annual household income was considered appropriate because income status of households not only depends on the number of wage earners but also on type of occupation and possession of income-generating assets. Information on income was not available (i.e., nonresponse) from four of the households surveyed.

**Table 4:** Results of a two-step cluster analysis of containers, statistics (percentage and mean ± SE) of some of the variables used in the classification, and pupae of *Aedes aegypti*, Dhaka, during the 2011 monsoon season

Derived clusters	Percentage of containers per cluster	Percentage of containers under the full shade (both indoor and outdoor)	Percentage of containers with rain water in them	Percentage of containers with vegetation nearby	*Ae. aegypti* pupae per container (mean ± SE)	Total count of pupae per cluster (% of pupae)
I	41.7	90.8	0.0	18.3	(0.94 ± 0.20)*	192 (34.9)
II	58.3	9.2	100.0	81.7	(1.25 ± 0.19)†	358 (65.1)

SE = standard error.

* Container sample size in cluster I was 206.

† Container sample size in cluster II was 287.

**Table 5:** Results of multivariable logistic regression model of socioeconomic/infrastructural factors associated with the possession of container categories (functional) by householders (*N* = 221), Dhaka, 2011

Explanatory variable	Container category	Estimates (SE)	*P* value	OR (95% CI)
Constants	B	−1.46 (0.57)	0.106	
	C	−1.44 (0.56)	0.009	
	D	−1.03 (0.47)	0.03	
	E	−1.23 (0.61)	0.043	
Income of the household*‡				
2 vs. 1	B vs. A	−0.40 (0.64)	0.53	0.66 (0.19–2.35)
2 vs. 1	C vs. A	0.01 (0.59)	0.99	1.00 (0.31–3.22)
2 vs. 1	D vs. A	0.04 (0.52)	0.944	1.04 (0.38–2.84)
2 vs. 1	E vs. A	−1.54 (0.86)	0.073	0.22 (0.04–1.16)
3 vs. 1	B vs. A	−1.02 (0.63)	0.105	0.36 (0.11–1.14)
3 vs. 1	C vs. A	−1.20 (0.65)	0.067	0.30 (0.08–1.09)
3 vs. 1	D vs. A	−1.09 (0.54)	0.042	0.33 (0.12–0.96)
3 vs. 1	E vs. A	−2.80 (1.11)	0.011	0.06 (0.01–0.53)
Purpose of storing water†§				
2 vs. 1	B vs. A	1.19 (0.55)	0.03	3.29 (1.11–9.71)
2 vs. 1	C vs. A	1.17 (0.53)	0.027	3.22 (1.14–9.10)
2 vs. 1	D vs. A	1.27 (0.44)	0.004	3.55 (1.48–8.49)
2 vs. 1	E vs. A	0.86 (0.70)	0.22	2.37 (0.60–9.34)

A = household chores; B = ornamental; C = amenities; CI = confidence interval; D = discarded; E = repairing and reconstruction; OR = odds ratio; SE = standard error.

* 1 = high (> 70K Taka); 2 = medium (30K–70K Taka); 3 = low (< 30K Taka).

† 1 = drinking and cooking; 2 = cleaning, washing, and showering.

‡ Overall χ^2^ value = 15.62, degrees of freedom (df) = 8, *P* = 0.0481.

§ Overall χ^2^ value = 11.82, df = 4, *P* = 0.018.
