# Supplementary material for: Socioeconomic and Ecological Factors Influencing Aedes aegypti Prevalence, Abundance, and Distribution in Dhaka, Bangladesh
Source: Am J Trop Med Hyg. 2016 Jun 1;94(6):1223–33. doi: 10.4269/ajtmh.15-0639 (PMC4889738; doi:10.4269/ajtmh.15-0639)
Supplement: Supplementary file 1 [file SD1.pdf]

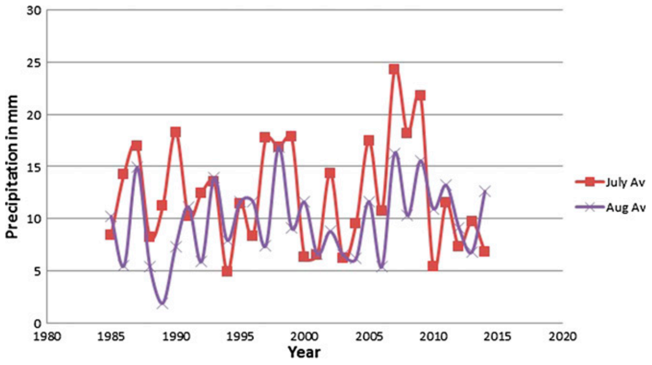

Supplemental Figure 1. Average precipitation in Dhaka for July and August, 1985–2014.

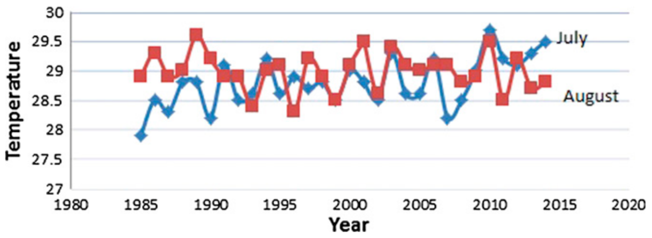

Supplemental Figure 2. Trend in average temperature in Dhaka for July and August, 1985–2014.

Supplemental Table 1

Number and percentage distribution of the most productive containers, and other positive containers in which *Aedes aegypti* or *Aedes albopictus* larvae and/or pupae was detected in households inspected in Dhaka, Bangladesh, 2011 monsoon season

Container type	Total container inspected (*N* = 1,250)	Positive containers (*N* = 493)	Percentage of containers with larvae and/or pupae
Most productive container			
Water tank	76	21	27.63
Abandoned plastic bucket	180	48	26.67
Plastic barrels (sealable)	148	48	32.43
Flower tub and tray	103	58	56.31
Tires	61	34	55.74
Clay pot	74	41	55.41
Disposable plastic container	90	36	40.00
Refrigerator trey	85	44	51.76
Plastic bottle	118	41	34.75
Other positive container			
Clay pitcher	8	2	25.00
Tin/metal can	14	3	21.43
Metal pan	9	4	44.44
Metal bucket	27	14	51.85
Plastic bags	28	7	25.00
Metal drum	20	5	25.00
Cement pot	24	13	54.17
Money plants tub	30	18	60.00
Coconut shell (dry or green)	22	6	27.27
Plant axil	8	4	50.00
Tree hole	5	4	80.00
Bamboo stamp	17	2	11.76
Battery shell	2	2	100.00
Plastic sheet to cover objects	18	9	50.00
Shorea robusta tree leaves	3	1	33.33
Ceramic pot	6	5	83.33
Wood slab	1	1	100.00
Aluminum pot	22	5	22.73
Glass bottle	11	3	27.27
Plastic mug	31	10	32.26
Earthen jar ("motka")	9	4	44.44

Supplemental Table 2

Percentage distribution of most productive containers and most abundant containers infested with *Aedes aegypti* or *Aedes albopictus* larvae and/or pupae in households inspected in Dhaka, Bangladesh, 2011 monsoon season

Container type	*N*	%	Container type	*N*	%
Most productive containers			Most abundant containers		
Temporary water tank	186	4.41	"Money plant" tub	142	3.36
Plastic bottle	329	7.79	Disposable plastic container	335	7.94
Disposable plastic container	335	7.94	Clay pot	342	8.10
Clay pot	342	8.10	Abandoned plastic bucket	366	8.67
Abandoned plastic bucket	366	8.67	Plastic barrels (sealable)	393	9.31
Plastic barrels (sealable)	393	9.31	Refrigerator tray	396	9.38
Refrigerator tray	396	9.38	Flower tub and tray	470	11.13
Flower tub and tray	470	11.13	Tires	474	11.23
Tires	474	11.23			
Subtotal (most productive containers)	3,291	77.97	Subtotal (most abundant containers)	2,918	69.13
Other remaining productive containers (*N* = 21 types)	930	22.03		1,303	30.87
Total (all containers)	4,221	100.00		4,221	100.00
